# Identifying Nutritional Insecurity Among Families in an Urban Pediatric Practice

**DOI:** 10.1001/jamanetworkopen.2023.1709

**Published:** 2023-03-06

**Authors:** Yonit Lax, Danielle Cullen, Michael Silver, Jeffrey R. Avner

**Affiliations:** 1 Maimonides Medical Center, Brooklyn, New York; 2 SUNY Downstate Health Sciences University, Brooklyn, New York; 3 Children’s Hospital of Philadelphia, Philadelphia, Pennsylvania; 4 University of Pennsylvania Perelman School of Medicine, Philadelphia

## Abstract

This cross-sectional study examines the prevalence of and concordance between self-reported food scarcity and nutritional insecurity in an urban pediatric practice.

## Introduction

Food insecurity (FI) is defined as the state of being without reliable access to a sufficient quantity of affordable, nutritious foods.^1^ Although this definition includes nutritious foods, FI screening has largely focused on quantity and not the quality of food.^2,3^ Families with FI often depend on calorie-dense, nutrient-poor foods because of barriers related to cost and access.^4^ Therefore, families may experience nutritional insecurity (NI), defined as inconsistent access, availability, and affordability of food that promotes well-being,^3^ as a last safeguard against food scarcity (FS), which is defined as limited access to adequate food. This cross-sectional study aimed to (1) determine the prevalence of self-reported FS and NI in an urban pediatric practice and (2) evaluate the concordance between FS and NI.

## Methods

In April 2019, an electronic health record–based social risk screening and referral model that included 1 question on FS and 1 question on NI was implemented in 3 urban pediatric primary care sites (Maimonides Pediatrics at 57th Street, Brooklyn, New York; Maimonides Newkirk Family Health Center, Brooklyn, New York; and Maimonides Pediatrics at 7th Avenue, Brooklyn, New York). The screening tool uses an adapted version of the first question in the American Academy of Pediatrics toolkit,^5^ for which an affirmative response to this question alone is considered an indicator for FI and was found to have a high sensitivity. The question asked, “In the last 12 months, did your family worry whether your food would run out before you got money or SNAP/food stamps to buy more?” The second question inquired about access to nutritious foods: “Within the past 12 months, did you have enough money to buy fruits and vegetables to feed your family nutritious foods?” Race and ethnicity were self-identified by the participants and were assessed in the study to further understand the needs of the different populations we serve. A cross-sectional descriptive study was conducted with electronic health record data from April 2019 to October 2021.

The Maimonides Medical Center institutional review board deemed this study exempt from review and waived the requirement for informed consent because it was conducted as a quality improvement measure as part of health care operations, in accordance with 45 CFR §46. This study followed the Strengthening the Reporting of Observational Studies in Epidemiology (STROBE) reporting guidelines.

Data analysis was conducted from October 2021 to January 2023 with SPSS statistical software version 28 (IBM). Statistical significance was set at 2-sided α = .05 (analysis of variance or Fisher exact test).

## Results

Of the 14 926 pediatric caregivers surveyed for FI, 6528 (43.7%) were non-English speakers, 6672 (44.7%) identified as Hispanic or Latino, and 11 502 (77.1%) were insured with Medicaid or Medicaid managed care. In total, 2786 (19.0%) screened positive for either FS or NI. Of these caregivers, 2043 (73.3%) reported NI and 1850 (66.4%) reported FS; 716 (25.7%) had FS alone, 909 (32.6%) had NI alone, and 1134 (40.7%) had both FS and NI (Figure). Families with NI alone were more likely than families with FS alone to have older children (mean age of children, 8.43 years [95% CI, 8.10-8.80 years] vs 7.53 years [95% CI, 7.10-7.90 years]; *P* = .005) and to have male children (497 male children [54.7%] vs 344 male children [48.0%]; *P* = .001, Fisher exact test), and were less likely to be Spanish speaking (491 participants [54.0%] vs 442 participants [61.7%]; *P* < .001, Fisher exact test) (Table).

**Figure.**
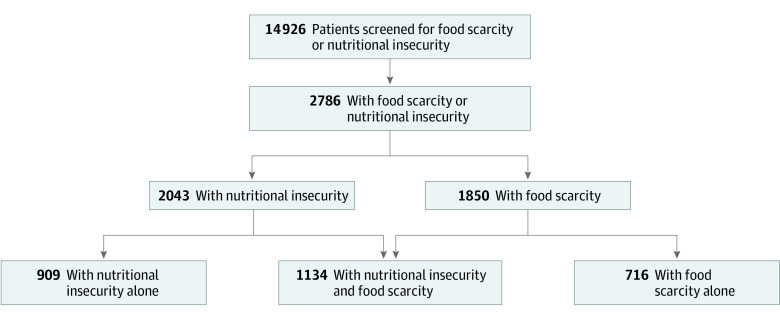
Food Scarcity and Nutritional Insecurity Participant Screening Flowchart

**Table.** Demographic Characteristics of Children With Food Scarcity, Nutritional Insecurity, and Both^a^

Variable	Children, No. (%)			*P* value
	Food scarcity only (n = 716)	Nutritional insecurity only (n = 909)	Both food scarcity and nutritional insecurity (n = 1134)	
Age, mean (95% CI), y	7.53 (7.10-7.90)	8.43 (8.10-8.80)	8.00 (7.70-8.30)	.005
Sex				
Female	372 (52.0)	412 (45.3)	603 (53.2)	.001
Male	344 (48.0)	497 (54.7)	531 (46.8)	
Race				
Asian	64 (8.9)	104 (11.4)	69 (6.1)	.003
Black or African	42 (5.9)	62 (6.8)	68 (6.0)	
Hispanic^b^	5 (0.7)	9 (1.0)	6 (0.5)	
White	221 (30.9)	278 (30.6)	327 (28.8)	
Adult respondent declined to answer	9 (1.3)	10 (1.1)	13 (1.1)	
Unknown	22 (3.1)	38 (4.2)	52 (4.6)	
Other^c^	353 (49.3)	408 (44.9)	599 (52.8)	
Ethnicity				
Hispanic or Latino	464 (64.8)	573 (63.0)	751 (66.2)	<.001
Not Hispanic or Latino	112 (15.6)	193 (21.2)	161 (14.2)	
Unknown	140 (19.6)	143 (15.7)	222 (19.6)	
Language				
Bengali	16 (2.2)	24 (2.6)	24 (2.1)	<.001
Chinese	4 (0.6)	11 (1.2)	6 (0.5)	
English	237 (33.1)	362 (39.8)	314 (27.7)	
Spanish	442 (61.7)	491 (54.0)	774 (68.3)	
Other	17 (2.4)	21 (2.3)	16 (1.4)	
Payer				
Child Health Plus	8 (1.1)	16 (1.8)	27 (2.4)	.002
Commercial	15 (2.1)	42 (4.6)	19 (1.7)	
Medicaid or Medicaid	614 (85.8)	760 (83.6)	968 (85.4)	
Self-pay	79 (11.0)	91 (10.0)	120 (10.6)	

^a^ Age was summarized with mean and 95% CI and compared across groups using analysis of variance. All other variables were summarized with frequency and percentage and compared across groups with a Fisher exact test.

^b^ Some respondents chose to identify Hispanic as their race rather than ethnicity.

^c^ Other race includes American Indian or Alaskan Native, Guamian, Chamarro, Maldivian, Middle Eastern, and other (adult respondent defined).

## Discussion

This cross-sectional study found that by screening using only questions related to FS, 32.6% of caregivers (909 of 2786 participants) with NI alone would have been missed. Thus, conventional screening for FS may provide an incomplete picture, missing an important subset of patients who have NI. Evaluating for NI may be a valuable addition to FI screening, demonstrating financial strain and the use of coping strategies to avoid FS. This addition may provide an important opportunity for intervention with the advancement of food-as-medicine models, colocated community-supported agriculture programs, and opportunities to leverage Supplemental Nutritional Assistance Program and Women, Infants, and Children added produce benefits.^3,6^ Intervening at the start of FS when families are first experiencing NI may not only improve diet quality, but also help avoid the development of FI. Study limitations include the use of a nonvalidated question for NI, because no validated question existed at the time. In conclusion, practitioners should consider screening for both NI and FS. Further research is needed to validate a brief screening tool that evaluates for both NI and FS and considers family perceptions regarding these questions to help inform optimal screening results.

## Notes

Supplement.
Data Sharing Statement
