# Supplementary material for: Identifying Nutritional Insecurity Among Families in an Urban Pediatric Practice
Source: JAMA Netw Open. 2023 Mar 6;6(3):e231709. doi: 10.1001/jamanetworkopen.2023.1709 (PMC9989895; doi:10.1001/jamanetworkopen.2023.1709)
Supplement: Supplement. — Data Sharing Statement [file jamanetwopen-e231709-s001.pdf]

Data Sharing Statement

Lax. Identifying Nutritional Insecurity Among Families in an Urban Pediatric Practice. *JAMA Netw Open*. Published March 06, 2023. doi:10.1001/jamanetworkopen.2023.1709

Data
Data available: Yes
Data types: Deidentified participant data
How to access data: Ylax@maimonidesmed.org
When available: With publication

Supporting Documents
Document types: None

Additional Information
Who can access the data: Anyone requesting
Types of analyses: For any purpose
Mechanisms of data availability: With investigator support
